# Adolescent idiopathic scoliosis detection and referral trends: impact treatment options

**DOI:** 10.1007/s43390-020-00182-6

**Published:** 2020-08-11

**Authors:** Alison Anthony, Reinhard Zeller, Cathy Evans, Jennifer A. Dermott

**Affiliations:** 1 Division of Rehabilitation Services, Hospital for Sick Children, Toronto, ON Canada; 2 Division of Orthopaedic Surgery, Hospital for Sick Children, Toronto, ON Canada; 3 Department of Surgery, Faculty of Medicine, University of Toronto, Toronto, ON Canada; 4 Department of Physical Therapy, University of Toronto, 500 University Avenue, Toronto, ON M5G 1V7 Canada

**Keywords:** Adolescent idiopathic scoliosis, Pediatric, Appropriateness of care, Brace treatment, Referral pattern

## Abstract

**Study design:**

Retrospective cross-sectional study.

**Objective:**

To analyze the patient demographic referred for scoliosis to the Hospital for Sick Children to determine the proportion of patients suitable for brace treatment, as per the Scoliosis Research Society guidelines.

**Summary of background data:**

There is level 1 evidence that bracing in adolescent idiopathic scoliosis (AIS) decreases the risk of curve progression and need for surgery, but optimal brace treatment requires early curve detection.

**Methods:**

We performed a retrospective review of 618 consecutive patients who underwent initial assessment in our Spine Clinic between Jan. 1 and Dec. 31, 2014. We included children 10–18 years, with scoliosis greater than 10°, excluding those diagnosed with non-idiopathic curves. Primary outcomes were Cobb angle, menarchal status, and Risser score. We analyzed the effect of specific referral variables (family history, the person who first noticed the curve, and geographic location of residence) on presenting curve magnitude.

**Results:**

During the study period, 335 children met the inclusion criteria, with an average age of 14.1 ± 1.8 years and a mean Cobb angle of 36.8 ± 14.5°. Brace treatment was indicated in 17% of patients; 18% had curves beyond optimal curve range for bracing (> 40°), and 55% were skeletally mature, therefore not brace candidates. The majority of curves (54%) were first detected by the patient or family member and averaged 7° more than curves first detected by a physician. A family history of scoliosis made no difference to curve magnitude, nor did geographic location of residence.

**Conclusion:**

The majority of AIS patients present too late for effective management with bracing.

**Level of evidence:**

III.

## Introduction

Adolescent Idiopathic Scoliosis (AIS) is the most common form of scoliosis. It occurs in otherwise healthy children around puberty, during the period of rapid skeletal growth [1–4]. Scoliosis is a structural deformity of the spine characterized by vertebral rotation and a lateral curvature measuring greater than 10° [1, 2, 5]. Eighty percent of scoliosis curves are considered idiopathic [1, 4] with the remaining curves secondary to a known pathological process (e.g., associated with a neuromuscular disorder or congenital vertebral anomaly). The prevalence of AIS is 1–3% in the general population with a higher prevalence (approximately 6:1) seen in females, [1, 6]. Female, large curve magnitude at initial presentation, and skeletal immaturity (particularly pre-menarchal status) are all independent risk factors for curve progression [7–13].

To standardize scoliosis treatment, the Scoliosis Research Society (SRS) [13] has developed guidelines based on the magnitude of curvature, described by the Cobb angle [14, 15], and skeletal growth potential, using the Risser Scale [15, 16]. The SRS treatment recommendations for AIS fall into three main categories: observation, bracing, and surgery [17]. For patients with curves between 11 and 24° and spine growth remaining (Risser 0–2), clinical and radiographic observations are recommended approximately every 4–6 months to monitor possible curve progression [11, 13]. Rigid, spinal bracing is recommended for growing children with curves between 25–40°. Surgery, by way of posterior spinal fusion (PSF), is indicated with more severe curves [1, 13]. The SRS guidelines consider curves between 40 and 50° borderline, or a “gray zone” for surgical consideration although many centers, including ours, use greater than 50° as the threshold for surgery [11, 17].

Bracing using rigid, spinal orthoses minimizes the risk of curve progression in skeletally immature patients, reducing the likelihood of requiring surgery [4, 7, 11, 13]. Through a multicentre randomized clinical trial, commonly referred to as the BrAIST study, Weinstein et al. (2013) showed that, in a cohort of 242 patients with growth potential remaining, 72% of braced patients avoided surgical recommendations (defined as 50°) compared to only 48% of observed patients [7]. Upon skeletal maturity, those with curves less than 30° are considered stable, whereas curves greater than 30° have an increased risk of continuing to progress. Specifically, Weinstein and Ponseti (1983) found that over a 40-year period, thoracic curves between 30° and 50° showed an average progression of 10° and between 50° and 75° showed an average progression of 29° [18]. This provides rationale for aiming to keep curves below 50°, and highlights the importance of early curve detection and timely brace prescription. Late referrals are those children that are likely to require surgery; those that continue to have growth potential but present with a curve beyond the range that brace treatment is considered most effective for children (i.e., greater than 40°), and growing or not, that present with curves greater than 50°. Presently, the spine program at our publicly funded pediatric tertiary care institution is under-pressure to meet the demand for scoliosis surgery with an average 24-month waitlist. This study aims to elucidate the extent to which late AIS referrals and the missed opportunity for brace management may ultimately be contributing to lengthy surgical lists.

The primary objective of this study was to characterize the profile of children with AIS upon their initial assessment to our center to quantify (1) the number of children suitable for brace treatment and (2) the number of children that were referred late. Second, we analyzed the effect of referral variables on Cobb angle at initial presentation to see if the magnitude of the curve was influenced by where a patient lives, based on their Local Health Integration Network (LHIN), a family history of scoliosis, or who first detected the curve. We hypothesized that: patients living a greater distance from our center are at higher risk for presenting late given that there are less primary care physicians per capita and the travel distance to reach a physician is greater, those with a positive immediate family history are at lower risk as they may be more closely monitored, and based on our clinical experience, the majority of curvatures are first recognized by a non-medical individual. The study results will inform the direction of and serve as a comparative baseline for future quality improvement initiatives to enhance the early detection and timely referral of AIS patients, at the institution and community level, with the overall goal being to minimize the number of AIS patients that ultimately require surgery.

## Methods

### Setting

**Fig. 1:**
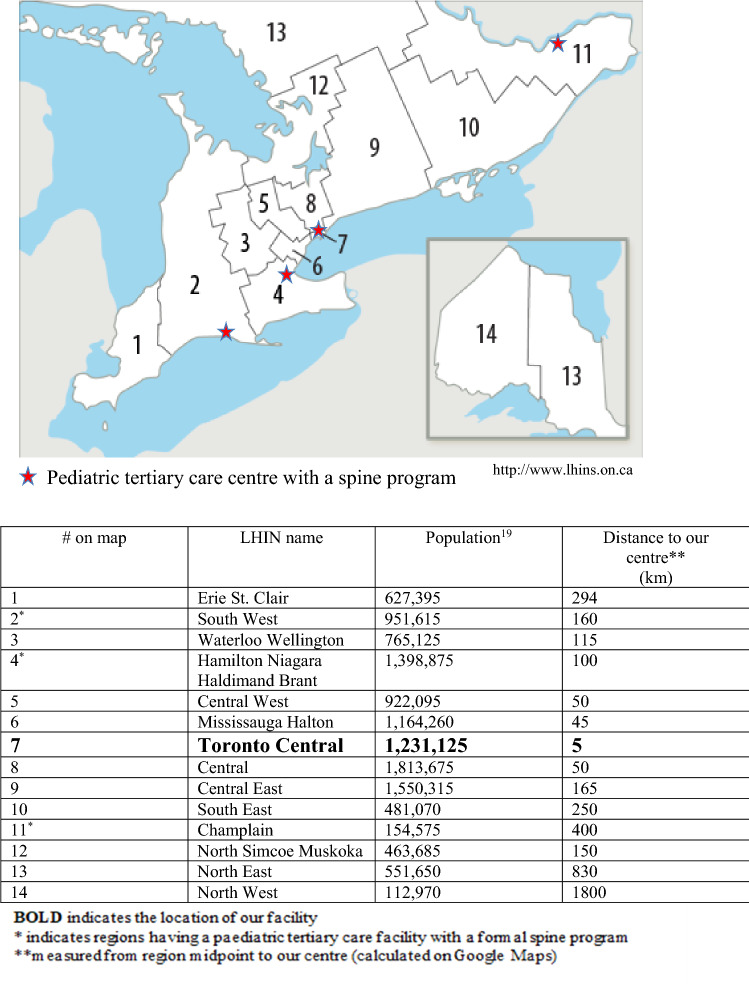
Local Health Integration Networks in Ontario

At the time of the study, the Spine Clinic at the Hospital for Sick Children, a pediatric tertiary care, university affiliated center, was staffed with 1.2 orthopedic pediatric spine surgeons, an orthopedic spine fellow, 2 nurse practitioners, and a physical therapy practitioner. This clinic receives the largest proportion of spine related referrals in Ontario. Ontario is 1.076 million km^2^ with a population of 13.6 million people [19] and is divided into 14 LHINs (see Fig. 1). These are community-based, government funded, non-profit organizations that coordinate services delivered by the hospitals and community care facilities for a specific geographic region [20]. Our institution averages over 800 referrals per year for spinal pathology, with approximately 40% of patients diagnosed with AIS. Children who are skeletally immature (i.e., based on Risser staging and/or menarchal status for females) are triaged and seen for an initial consultation within 6 weeks of our center receiving the referral. Referrals deemed low risk for significant progression are seen within 3 months.

### Study design and population

**Fig. 2:**
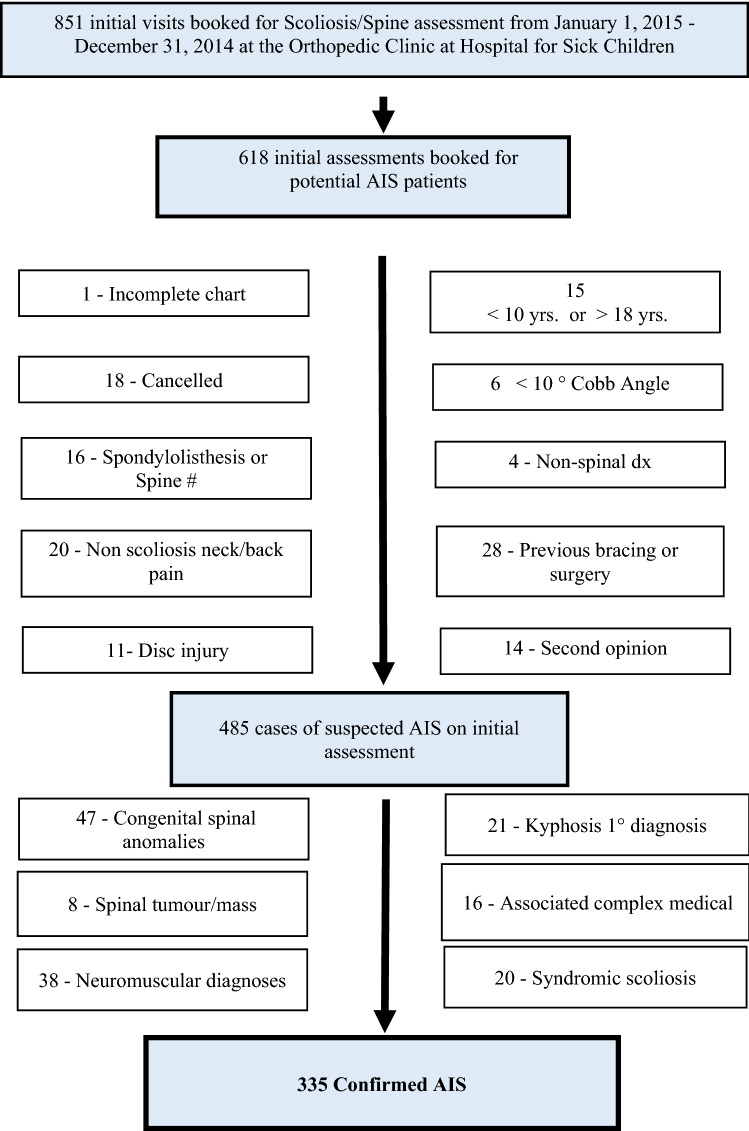
Classification of suspected adolescent idiopathic scoliosis cases according to defined criteria of appropriateness of referral

We conducted a cross-sectional, retrospective cohort study in accordance with the hospital and the university Research Ethics Board-approved protocol. We reviewed the health care record and spinal radiographs for all children, between 10 and 18 years of age who had an initial assessment in our Spine Clinic from January 1 to December 31, 2014. A full year was selected to ensure comprehensive representation of the population, limiting the effect of seasonal influences. Exclusions included those with a spinal curve less than 10°, a non-scoliosis spinal diagnosis (e.g., # vertebrae), a non-AIS diagnosis (e.g., neuromuscular or syndromic scoliosis), a history of previous spinal surgery/bracing, or were seeking a second opinion (Fig. 2).

### Outcomes

The primary outcomes pertained to clinical and radiographic presentation. The Cobb angle was recorded for an individual’s largest curve. The type of curve (i.e., thoracic, thoracolumbar, and lumbar) was determined by the level of the apex of the curve [15]. The Risser score [16] was noted. Menarchal status was categorized based on time passed from onset of menses to date of referral (pre-menarchal, < 6 months, 6 to < 12 months, 12 to < 18 months, 18 to < 24 months, and ≥ 2 years). Secondary outcomes focused on referral variables that could be extracted by chart review: the person that first noticed the curve, reported family history of scoliosis (first degree relative), and the geographical area of residence (LHIN).

### Statistical analyses

Descriptive statistics (mean, standard deviation [SD], range, and frequencies) pertaining to Cobb angle were calculated for the entire cohort and separately by gender, curve type, and for females, menarchal status. Frequencies were calculated for categorical data. Independent *t* tests were performed on continuous data and chi-squared tests on categorical data to compare clinical presentation by gender. Analysis of variance was used to assess Cobb angle by geographic location, family history, and the individual first identifying the curve, with Tukey’s method applied when Cobb angle differed significantly by sub-group. Statistical significance was accepted at a *p* value less than 0.05. Data were analyzed using the web-based stats program R (www.R-project.org) [21].

### Results

A total of 335 patients were eligible for inclusion in the study (Fig. 2) with an average age of 14.1 ± 1.8 years (range 10.0–17.8 years) and a mean Cobb angle of 36.8° ± 14.5° (range 10°–88°). Thoracic curves were the most common (58.5%) and had the largest mean Cobb angle at 39.7° ± 14.9° (Table 1). Females accounted for 79% (*n* = 265) of the patient group with the majority were either pre-menarchal (31.3%) or 2 years post-menarchal (32.8%) (Table 2). Males were older than females at initial presentation (15.1 vs. 13.9 years, *p* < 0.05) however; both genders were comparable in Cobb angle (*p* = 0.16), curve type, and Risser score (*p* = 0.99) (Table 1).

**Table 1:** Comparing age, curve size/type, and skeletal maturity between male and female patients with AIS on initial assessment to spine clinic

	Female (*n* = 265)	Male (*n* = 70)	Total (*n* = 335)	*p* value
Age, mean ± SD, year	13.9 ± 1.7	15.1 ± 1.6	14.1 ± 1.8	3.28e−07*
Cobb angle, mean ± SD °	37.4 ± 14.1	34.5 ± 15.7	36.8 ± 14.5	0.16
Curve type, *n* (%)				0.99
Thoracic (mean Cobb angle 39.7° ± 14.9°)	155 (58.4)	41 (58.6)	196 (58.5)	
Thoracolumbar (mean Cobb angle 33.9° ± 12.8°)	62 (23.4)	17 (24.3)	79 (23.6)	
Lumbar (mean Cobb angle 31.0° ± 13.1°)	48 (18.1)	12 (17.1)	60 (17.9)	
Risser sign, *n* (%)				0.99
0	67 (25.3)	13 (18.6)	80 (23.9)	
1	22 (8.3)	9 (12.9)	31 (9.3)	
2	29 (10.9)	9 (12.9)	38 (11.3)	
3	23 (8.7)	7 (10.0)	30 (9.0)	
4	79 (29.8)	19 (27.1)	98 (29.3)	
5	45 (17.0)	13 (18.6)	58 (17.3)	

*SD* standard deviation

*Significant

**Table 2:** Menarchal status and mean Cobb angle at initial presentation to spine clinic

Menarchal status (missing 3) *n* = 262	*n* (%)	Mean Cobb angle ° ± SD
Pre-menarchal	82 (31.3)	36.5 ± 13.5
< 6 months post-onset of menses	26 (9.9)	39.6 ± 14.6
6 < 12 months post	26 (9.9)	38.7 ± 15.5
12 < 18 months post	21 (8.0)	41.2 ± 18.0
18 < 24 months post	21 (8.0)	41.7 ± 16.6
> 2 years post	86 (32.8)	35.3 ± 12.5

*SD* standard deviation

*No significance in Cobb angle by menarchal status *p* = 0.29

**Fig. 3:**
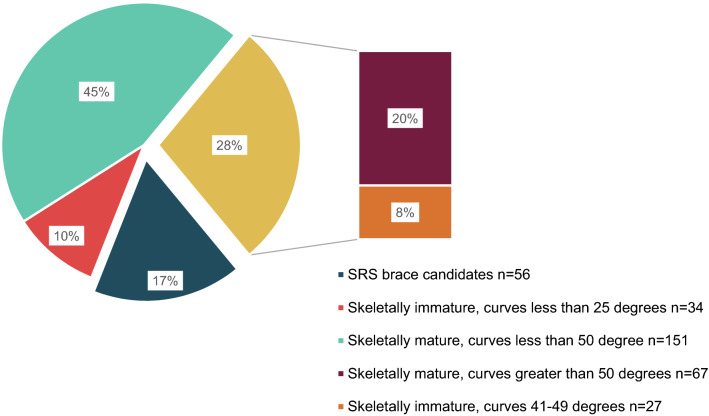
Distribution of adolescent idiopathic scoliosis patients presenting to spine clinic for initial assessment

**Fig. 4:**
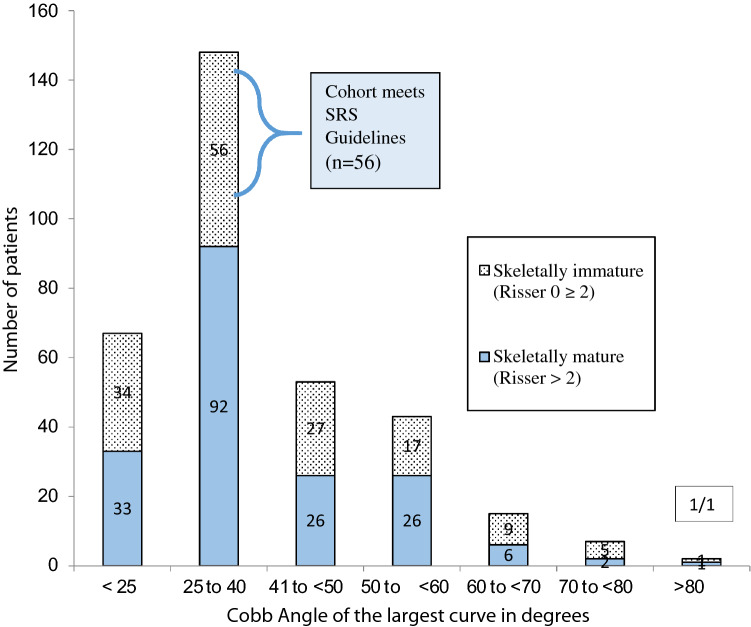
Relationship between Cobb angle and level of skeletal maturity of adolescent idiopathic scoliosis patients presenting to spine clinic on initial assessment

Seventeen percent of the cohort (*n* = 56) met SRS guidelines for bracing (Cobb angle 25°–40°, Risser score 0–2), while 67 adolescents (20%) presented with a curve magnitude above the threshold for surgical consideration (> 50°) (Fig. 3). A further 27 patients (8%) were at high risk for requiring future surgery as their curve magnitude exceeded brace indications (Cobb angle 41–49°) and they continued to have growth potential remaining (Fig. 3). Figure 4 illustrates the distribution of patients by curve magnitude and shows that more than half (*n* = 186, 55%) were at or near skeletally mature (Risser > 2).

In 237 charts (71%), the person that first identified the curve was recorded. A non-medical layperson was the first to observe the curve in 59% of patients (patient/family = 54%, non-medical individual such as coach = 5%), whereas 41% of curves were first detected by a medical professional (physician = 33%, allied health professionals = 8%). Individuals whose scoliosis was first recognized by a family physician/pediatrician had a mean curve at the initial presentation 7.1° less (*p* = 0.01) than those first noticed by the patient or their family member (Table 3).

**Table 3:** Referral variables at presentation to spine clinic

Characteristics	*n* (%)	Mean Cobb angle (°)
Family history (missing: 81, 2 adopted and unknown) (*p* = 0.75)		
Yes	72 (28.3)	35.6 ± 13.5
No	182 (71.7)	36.2 ± 13.8
Who first noticed? (missing: 98)		
Physician/pediatrician	70 (29.5)	34.3 ± 12.6
Other medical specialist	8 (3.4)	27.9 ± 11.6
Physical therapist	6 (2.5)	35.0 ± 4.8
Other health care professional	14 (5.5)	40.8 ± 15.7
Family/patient	127 (53.6)	41.9 ± 14.9^a^
Non-medical individual	12 (5.1)	38.9 ± 18.2
LHIN (*n* = 335)		
Erie St. Clair	0 (0)	
South West*	1 (0.3)	31.0
Waterloo Wellington	4 (1.2)	40.8 ± 6.1
Hamilton Niagara Haldimand Brant*	2 (0.6)	78.5 ± 13.4^b^
Central West	47 (14.0)	38.3 ± 14.6
Mississauga Halton	45 (13.4)	40.0 ± 14.3
Toronto Central*	**68 (20.3)**	**33.2 ± 12.4**
Central	112 (33.4)	34.2 ± 14.3
Central East	31 (9.3)	40.1 ± 14.5
South East	2 (0.6)	51.5 ± 2.1
Champlain*	0 (0)	
North Simcoe/Muskoka	16 (4.8)	44.4 ± 15.3
North East	7 (2.1)	33.7 ± 14.1
North West	0 (0)	

Bold indicates the location of our facility

*Indicates regions having a pediatric tertiary care facility with a formal spine program

^a,b^Tukey multiple comparisons of means demonstrated significant difference (*p* adj < 0.05) in Cobb angle between ^a^family/patient versus physician/pediatrician (*p* = 0.011), and ^b^Hamilton Niagara Haldimand Brant versus Central, Central East, Central West, Mississauga Halton, North East, and Toronto Central

Adolescents with a reported family history of scoliosis (28%) did not present with a significantly different Cobb angle (*p* = 0.75) at the initial assessment. There was no significant difference in mean Cobb angle at the initial assessment based on geographic location of residence (LHIN) with one exception (Table 3). Of note, there is a tertiary care facility within that LHIN that provides management for scoliosis patients with mild-to-moderate curves.

## Discussion

Brace treatment is a conservative management modality that aims to decrease the burden of operative treatment for AIS patients. Despite high-level evidence supporting the use of bracing to minimize the risk of scoliosis curve progression to surgical range [7], our study highlights that at the initial presentation, only a small number of patients (*n* = 56/335, 17%) are suitable for brace treatment, as per the SRS guidelines [17]. Furthermore, a greater number of patients (*n* = 67, 20%) present with large magnitude curves already in range for surgical consideration. Contrary to our a priori hypotheses living further from our center or having a family member with scoliosis did not correlate to curve magnitude at initial presentation. As expected, the majority of curvatures were first detected by a non-medical lay person.

Most studies define a late referral as a failure to recognize a curvature early enough to initiate brace treatment [22]. Most patients that are skeletally mature at initial presentation do not have curves large enough to require surgery; however, their curve magnitude may be larger than it would have otherwise been if they had undergone brace treatment. There is little empirical evidence that the magnitude of a non-surgical curve correlates with health-related quality of life [23]. The late referrals of greatest concern are those that may have avoided surgery had their curves been detected earlier when brace treatment was a reasonable option (i.e., curves less than 40°). In our study, 94 adolescents are likely to require a PSF, 27 patients that were continuing to grow but had curves over 40° and a further 67 patients with curves greater than 50° at the initial presentation (Fig. 3). For AIS curves prescribed a brace between 20° and 40°, Risser 2 or less, the BrAIST study demonstrated a 1.93 adjusted odds ratio of not progressing to greater than 50°. Applying this to our 94 late referrals suggests that, had they been referred in time for brace treatment, 61 patients may have avoided likely PSF. These patients would not be subject to the risks associated with this highly invasive procedure, such as surgical site infection, neurologic injury, and/or implant complications. Nor would they face the disruption of a lengthy recovery that may necessitate time away from friends, school, and extra-curricular activity. Aside from the implications to the individual patient, this sizeable volume places a consequential burden on the healthcare system and the capacity of an institution’s resources. For a spine surgeon averaging 1.25 cases per week, over 90% of their surgeries performed each year would be related to the late referral of an AIS patient. This creates access challenges for other spinal deformity patients that require timely intervention, for example, symptomatic high-grade spondylolisthesis, severe neuromuscular or syndromic scoliosis, or progressive early onset scoliosis where casting or growth sparing techniques are indicated.

There does not appear to be any acute effect of the publication of the BrAIST study on early curve detection or the referral of patients given that our findings are consistent with the previous studies that show in a universal health care system with no school screening, the majority of new patients seen in a specialty scoliosis clinic are near or at skeletal maturity or have a Cobb angle greater than 40° and, therefore, are not brace candidates. In our cohort, 55% had limited growth potential and 18% had a curve magnitude greater than 40°. Similarly, Beausejour et al. [22] in Montreal, Canada found that 56% of their new patients were considered late referrals (41% had a Risser sign > 2; 16% with a curve > 40°) and Abodor et al. [24] reported that of the 60% referred late to their center in Norway (61% of new patients had a Risser sign of > 2 and 39% curves of > 40°). Both the Norway and Montreal studies compared AIS populations that had and had not been exposed to scoliosis screening. Adobor et al. [24] prospectively collected characteristics of patients with AIS at their centers from 2003 to 2011 (no screening) and compared these data with historical control data from similar patients seen between 1976 and 1988 (exposed to scoliosis screening). Their findings suggested more patients in the non-screened group were treated with surgery and fewer were managed with bracing. Similar to our study where the mean Cobb angle at the initial presentation was 36.8° ± 14.5°, they found the initial curve sizes in their group approached the upper limit of brace treatment indications. Montreal also noted that their late referral rate increased after the cessation of school screening.

Community screening programs in Canada were implemented in public schools in the early 1970s, but discontinued less than a decade later as they were considered too costly, with little high-quality evidence at that time to justify conservative intervention. School screening for scoliosis programs (SSSP) is no longer practiced in Canada. Given the evidence of brace efficacy in minimizing curve progression and the ultimate need for surgical intervention, there is a renewed interest in optimizing early detection and referral of AIS. The SRS, American Academy of Orthopedic Surgeons, Pediatric Orthopedic Society of North America, and the American Academy of Pediatrics recommend that girls be screened, by well-trained screening personnel, for scoliosis at ages 10 and 12 years and boys at either 13 or 14 years. Although the average curve magnitude at the initial presentation may be smaller in health care systems with SSSP, the increase in unnecessary radiographs ordered and heightened clinic volume demands must be considered. A meta-analysis estimating the effectiveness of SSSP indicates that it may not be performed effectively given its low positive predictive value (PVV) for detecting curves greater than 20° [25].

Presently Canada, the onus of diagnosing and referring scoliosis patients to a specialized orthopedic clinic falls on the primary care practitioner. They use their clinical judgment based on physical examination (commonly the forward bend test), to determine if an X-ray is warranted. We found that the family doctor/pediatrician first noticed the curve in only 30% of cases. One possible explanation is that not all children have access to a regular primary care physician and even less receive routine well-child visits. In 2012, 132 000 children in Ontario did not have a family doctor [26]. In Denmark, also a public health care system without SSSP, the average scoliosis curvature at initial assessment was 27° versus 37° in our study. The Danish Ministry of Health has published guidelines on the evaluation of a suspected spinal deformity for children which may contribute to earlier detection and referral of curves in this country [27]. There is no information available related to the clinical effectiveness (PPV, negative predictive value). We found that over half (58%) of curves were first recognized by a non-medical individual with the majority of curves (54%) identified by patients or family members. Scoliosis curves that were first noticed by the patient or family were 7.1° larger than those identified by a physician, a difference both statistically (*p* = 0.011) and clinically significant (Table 3). A similar trend was found in the UK, where a majority (63%) of new AIS cases were first detected by family or friends, versus 26% that were first diagnosed by a general practitioner, with an average curve angle of 54° versus 43°, respectively [28].

There is an imperative for an in-depth qualitative assessment to identify barriers to early detection and timely referral of scoliosis. This may include focus groups and one-on-one interviews with patients to learn more about one’s personal experience leading up to diagnosis and from diagnosis to initial assessment at a tertiary care spine program. Potential barriers may include: limited access to a primary health care provider, a low number of well-child annual examinations being performed, adolescents, and/or family not immediately seeking medical consultation when they identify trunk asymmetry, primary health care providers not identifying smaller curvatures, or being unfamiliar with referral guidelines and treatment options, therefore not making timely referrals. The first step to enable change is addressing the identified barriers. Ensuring that adolescents are screened during high-risk growth periods is important, but should not be considered exclusive only to the school system. The authors propose that a multi-pronged strategy is needed. First, an institution-led health communication initiative [29] aimed to increase adolescent and parent awareness of the physical signs of scoliosis may facilitate improved self- or home screening. Information dissemination should capitalize on the popularity of social media platforms and content sharing via social media accounts (patients, district school boards, youth sport organizations, etc.). Second, an outreach program to primary care providers emphasizing clinical screening and referral guidelines may better support their clinical practice. Proposed screening activity should routinely include a systematic visual inspection of the patient from behind to identify any evidence of trunk asymmetry. This includes shoulder balance, waistline asymmetry, and any evidence of rotation where one side of the back would appear more prominent than the other. The patient should be further assessed for evidence of rotation using the Adam’s forward bend test. Family physicians and pediatricians may also use their screening as a teaching opportunity, explaining to the family and patient what they are looking for and what this may be evidence of. Family physicians and pediatricians may also use their screening as a teaching opportunity, explaining to the family and patient what they are looking for and what this may be evidence of. This may serve to increase awareness of the clinical manifestations of scoliosis, and promote self-assessment and the need to seek timely medical advice if at any point in time the adolescent or family notes change. It is not uncommon, in our own experience, to hear that a family noted trunk asymmetry several months to even years prior to formal diagnosis but attributed this to poor posture and believed that it would improve over time.

In response to this study, our institution has implemented a patient registry of pediatric spinal pathology that allows us to prospectively collect and analyze information pertaining to referral trends and subsequent treatment outcomes.

### Limitations

This study was challenged with the methodological limitations of retrospective data. Data obtained from subjective report from the family (i.e., who first noticed the curve, menarchal status, family history) were susceptible to recall bias. Data on measurement tools for identifying a curve were not collected. Some data were incomplete on the health record (family history, who first notice curve), although no more than 30%; nevertheless, calculations based on available data were considered adequate to be reflective of the population. Data were collected only from the initial assessment; therefore, we cannot speculate on the long-term outcome of these patients. We are unable to comment on the timeliness of diagnosis and/or referral related to patients previously treated elsewhere. They were excluded from our study as their initial assessment status was not available.

## Conclusion

Surgical waitlists are inflated by late AIS referrals. Some of these patients may have avoided surgery with earlier detection and/or timelier referral. Our study found that the majority of AIS patients are referred too late for effective treatment with spinal bracing. Given the Level 1 evidence supporting brace efficacy in minimizing the risk of curve progression to surgical range, this is concerning. Further work is needed to identify barriers of early diagnosis and referral to specialist centers, with the aim of reducing the number of patients who ultimately require surgical intervention.
